# Awake fiberoptic intubation of a patient with severe multiple trauma in prone position: a case report

**DOI:** 10.1186/s12871-024-02636-0

**Published:** 2024-07-19

**Authors:** Jingli Yang, Feng Zou, Guoping Ma

**Affiliations:** 1 Department of Anaesthesia, Affiliated Pudong Hospital of Fudan University, Shanghai, China; 2 Department of Anaesthesia, Eastern District of Shanghai Sixth Renmin Hospital, Shanghai, China; 3 Department of Anaesthesia, Affiliated Shanghai Traditional Chinese Medicine-Integrated Hospital of Shanghai University of Traditional Chinese Medicine, Shanghai, China

**Keywords:** Awake fiberoptic intubation, Multiple trauma, Prone position

## Abstract

**Background:**

Fiberoptic-guided intubation is considered as “gold standard” of difficult airway management. Management of the airway in prone position in patients with severe trauma presenting with penetrating waist and hip injury poses a major challenge to the anesthesiologist.

**Case presentation:**

A man presented with severe multiple trauma and hemorrhagic shock as a result of an industrial accident with several deformed steel bars penetrating the left lower waist and hip. It was decided to schedule an exploratory laparotomy following extracting the deformed steel bars. Successful administration of awake fiberoptic nasotracheal intubation, performed in a prone position under airway blocks and appropriate sedation, allowed for the procedure. The exploratory laparotomy revealed damage to multiple organs, which were repaired sequentially during a 7-hour surgical operation. The patient’s recovery was uneventful, and he was discharged from the hospital one month after the surgery.

**Conclusions:**

Awake fiberoptic nasotracheal intubation, along with airway blocks and appropriate sedation, can be a viable option in patients with severe multiple trauma in the prone position.

## Background

Difficult airway includes difficult mask ventilation, difficult intubation, or both. Fiberoptic-guided intubation is considered as “gold standard” of difficult airway management and has a definite place in the difficult airway algorithms of various professional bodies. Management of the airway in patients with severe multiple trauma presenting with penetrating waist and hip injury poses a major challenge to the anesthesiologist, who must ensure airway manipulation in the prone position avoiding the possible further injury in supine position. We herein present a case of an adult patient with severe multiple trauma who underwent awake fiberoptic nasotracheal intubation under airway blocks and sedation in the prone position.

## Case presentation

**Fig. 1:**
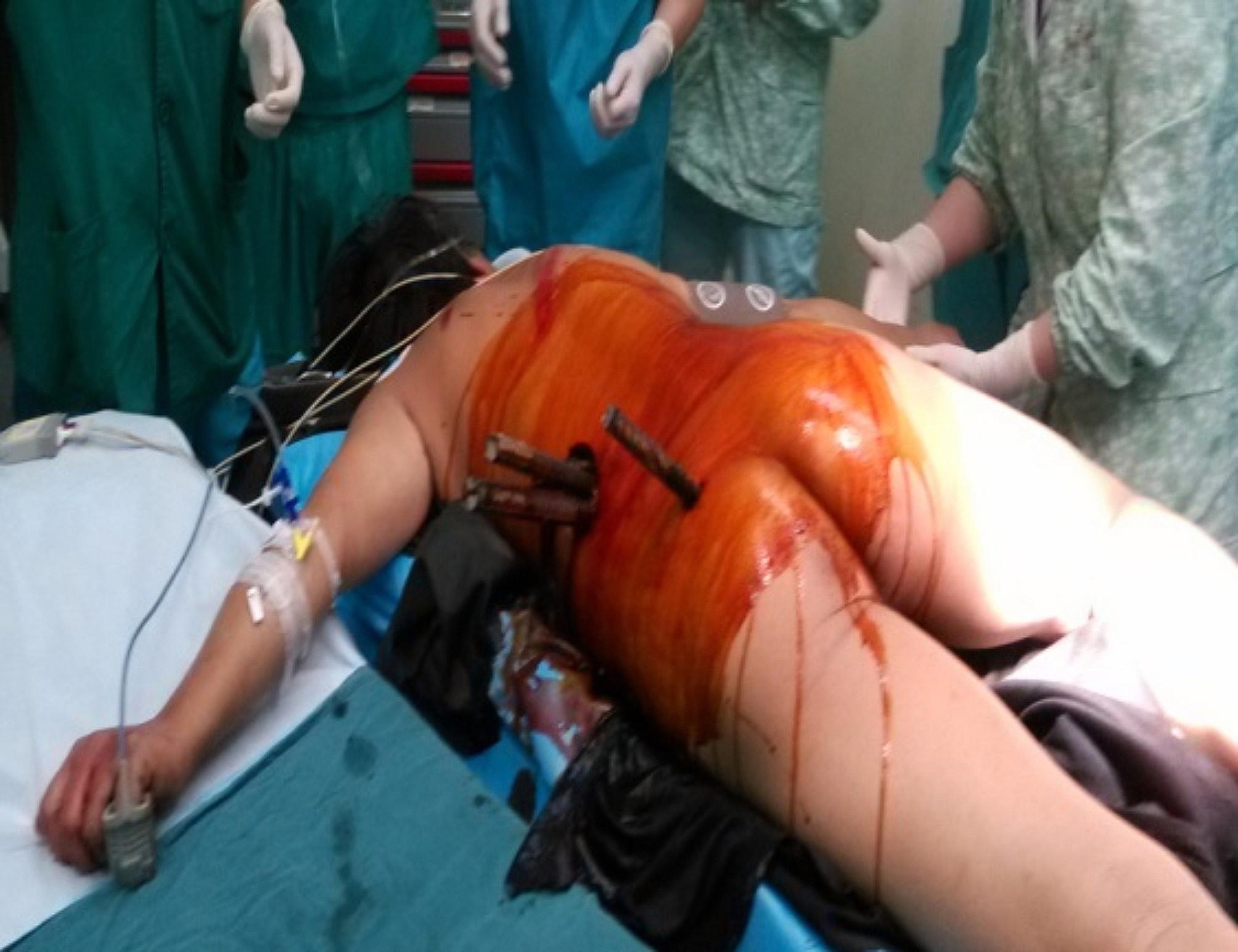
The patient in prone position

A 42-year-old man, in good health, standing at 168 cm and weighing 79 kg, was hospitalized due to multiple deformed steel bars piercing his left lower waist and hip. The injuries occurred as a result of an accidental fall and impact on his back and waist. The patient was promptly transferred to the central operating theater in a prone position through the designated “green channel” (Fig. 1).

Upon arrival at the central operating theater, the patient was conscious but displayed signs of pain, such as a distressed facial expression, pale eyelids, and cold, clammy limbs. He was breathing spontaneously, although decreased breath sounds were noted in the left lower lung lobe. His oxygen saturation level (SpO_2_) was 95%, and there were no signs of airway obstruction or abnormal head and neck movements. The patient’s hemodynamics appeared stable, with a blood pressure reading of 135/65 mmHg, a heart rate of 113 bpm, and a regular cardiac rhythm.

A computerized tomography (CT) scan revealed the presence of metallic foreign bodies in the abdominal and pelvic cavities, multiple fractures involving the ribs and the pubic symphysis, as well as indications of possible contusion in the liver, blood vessels, scrotum, and muscle groups. In light of these findings, a multidisciplinary team consultation was convened, and the patient was diagnosed with hemorrhagic shock and severe multiple trauma. The preliminary plan involved extracting the deformed steel bars and performing an exploratory laparotomy.

One of the main challenges in the perioperative period was performing emergency endotracheal intubation while the patient was in the prone position. This procedure needed to be carried out carefully to avoid further damage to organs and prevent exacerbation of the hemorrhagic shock during the extraction of the deformed steel bars.

**Fig. 2:**
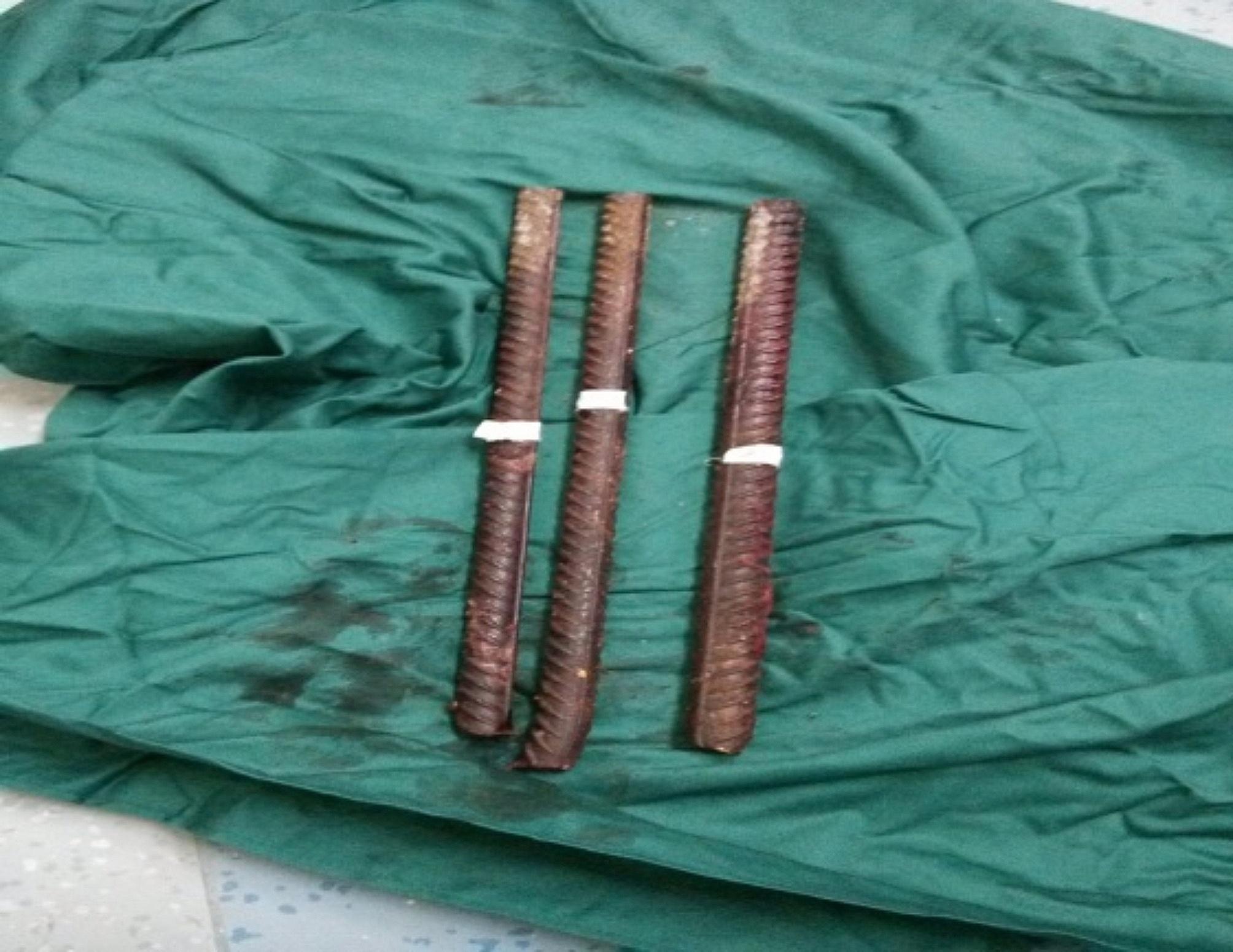
Three deformed steel bars penetrateted in the patient’s body

Since the patient was in a prone position and central venous catheterization was not feasible, two peripheral venous channels were established on the patient’s right upper and lower limbs respectively, upon transfer to the central operating theater. Another venous channel had been previously established in the emergency room. Considering the need to extract the deformed steel bars without causing pain or potential damage to abdominal and pelvic organs before anesthesia induction, our anesthesia team opted to establish the airway (tracheal intubation) while the patient remained in the prone position on operating table. The patient’s head was tilted to the right and extended beyond the operating table, with an anesthetist supporting it manually. Successful cricothyroid membrane puncture and spray with 1% tetracaine 1 ml were performed. Superficial anesthesia was achieved by spraying 1% tetracaine 1 ml on the floor of the mouth and the root of the tongue, while ephedrine 5 mg and 1% tetracaine 1 ml were carefully administered into both nasal cavities separately. Topicalisation was established and tested by an anesthetist, and another anesthetist helped him performing the fiberoptic intubation. After preoxygenation via nasal cannula with 6 L/min of oxygen for 5 min, the patient received sedation through an intravenous infusion of 5 µg of sulfentanyl and 1 mg of midazolam, aiming for a Ramesay score of 2–3. Fiberoptic intubation using a reinforced endotracheal tube (size 7.0) was successfully carried out through the right nasal cavity. The tube was first inserted blindly for a few centimeters and then the endoscope inserted over it in a second step. Once the flexible bronchoscope was in the trachea, and the carina was identified, the endotracheal tube was advanced continuously. Oxygen insufflation was performed via the working channel of the endoscope during the intubation process. Following confirmation of the proper placement of the endotracheal tube, additional doses of 2 mg of midazolam, 50 mg of propofol, 20 µg of sulfentanyl, and 50 mg of rocuronium were administered intravenously. The endotracheal tube was then held firmly in position and connected to the anesthetic breathing circuit, and mechanical ventilation was initiated. Anaesthesia was maintained using 2% sevoflurane in air/oxygen mixture with a flow rate of 1 L/min, while remifentanil and propofol were infused at rates of 0.1–0.15 µg·kg^− 1^·min^− 1^ and 50–150 µg·kg^− 1^·h^− 1^, respectively. Firstly, the deformed steel bars were extracted, and pledgets were used to pack the wound and control bleeding. Subsequently, the patient was repositioned into a supine position. A median abdominal incision was made for exploratory laparotomy following rapid disinfection and draping of the abdomen with a surgical sheet. Then nasopharyngeal temperature was monitored continuously, and a warm air blower was utilized to maintain the patient’s body temperature at 36–37℃. Since arterial blood gas analysis performed at the beginning of the operation revealed metabolic acidosis for HCO_3_^−^act 18mmol/L presented, a subsequent infusion of 100 ml of 5% sodium bicarbonate solution was administered. During the exploratory laparotomy, it was found that three deformed steel bars had penetrated the patient’s body to a depth of 12–15 cm (Fig. 2), causing damage to the liver, pancreas, stomach, spleen, and kidney. The surgical procedures included left nephrectomy and repair of the liver, pancreas, and stomach. Internal fixation was performed on the eighth, ninth, and tenth ribs. The duration of the surgery was 7 h, and the intraoperative blood loss amounted to 3000 ml. The fluid input consisted of crystalloid fluid, colloid fluid, calcium gluconate and blood components. Continuous infusion of norepinephrine was performed to prevent intraoperative hypotension. At the conclusion of the surgery, the urine volume was measured at 1500 ml, and another arterial blood gas analysis revealed normal acid-base balance and electrolytes.Despite of stabilization, considering the severity of the multiple trauma, the patient was transferred to the intensive care unit (ICU) without extubation immediately after the surgery. Extubation of the tracheal tube was performed on the second day after surgery when the patient regained consciousness and demonstrated stable respiratory and circulatory functions. The patient made a successful recovery and was discharged from the hospital one month post-surgery without any functional disorders in key organs.

## Discussion and conclusions

Awake fiberoptic intubation is widely recommended for managing anticipated or known difficult airways, particularly in patients with abnormal head and neck mobility and limited mouth opening [1]. The use of airway blocks and appropriate sedation during the procedure improves patient comfort, reduces airway reflexes, enhances patient cooperation and hemodynamic stability, and ensures airway preservation and spontaneous ventilation [2–4]. Similarly, a case report described awake fiberoptic intubation in a semi-prone position for a patient with facial trauma [5]. In this particular case, the patient could not be positioned supine due to penetrating deformed steel bars in the left lower waist and hip. Additionally, this patient was in shock and may have a compromised hemodynamics after induction of anaesthesia. Awake intubation using a flexible fiberoptic laryngoscope should be a safe and effective approach for airway management to the patient. After obtaining the patient’s medical history and conducting a physical examination, it was determined that there were no contraindications for nasotracheal intubation, making it the preferred option. Given the lack of access to cricothyroid membrane puncture and the patient’s severe traumatic pain and anxiety in the prone position, achieving optimal superficial anesthesia in the laryngopharynx and providing minimal sedation and analgesia were of utmost importance [4]. In this case, successful cricothyroid membrane puncture and spray were performed after extending the patient’s head and neck beyond the operating table. 1% tetracaine and ephedrine were sprayed for topicalisation, and that is not the same as recommendations by Difficult Airway Society (DAS).

However, the duration of anesthesia induction was slightly prolonged for a patient with hemorrhagic shock and severe multiple trauma. Comprehensive monitoring and evaluation during the perioperative period are crucial for assessing the condition and managing anesthesia in patients with severe trauma, as their condition is complex and rapidly progressing. In this particular case, a preoperative CT scan revealed multiple abdominal injuries, and the extraction of deformed steel bars had the potential to worsen these injuries and further exacerbate the patient’s condition. Therefore, after the extraction of the steel bars and turning the patient into a supine position, enhanced monitoring measures were implemented, including monitoring of central venous pressure (CVP), arterial blood pressure (ABP), bispectral index (BIS) and internal temperature.

The treatment of patients with severe multiple trauma is challenging, requiring a multidisciplinary and time-sensitive approach [6]. Therefore, the establishment of an effective multidisciplinary cooperation model for the management of these patients is of utmost importance.

The death triad includes metabolic acidosis in full blood, hypothermia, and coagulopathy when patients arrive at the emergency department, which observed in trauma patients has been proven to be a strong predictor of mortality [7–9]. Furthermore, hypothermia is also a significant contributor to coagulopathy, regardless of the condition of metabolic acidosis or fluid infusion [10, 11]. In this patient with severe trauma, we corrected the acidosis radically for a bicarbonate level of 18 to prevent the upcoming abnormal coagulation and poor prognosis. Additionally, measures to maintain proper body temperature were implemented. Hypotension, defined as an systolic blood pressure < 60 mmHg, may act as a proper death tetrad component to stratify the mortality risk of trauma patients, so continuous infusion of norepinephrine was performed to prevent post intubation hypotension. Though the surgery was successful, and the patient experienced a good recovery, a limitation of this report was that a point-of-care test(POCT) diagnostic was not performed timely to exclude abnormal coagulation function and hyperfibrinolysis as thromboelastography(TEG) was not avalible there in our hospital.

In conclusion, providing anesthesia to patients with severe multiple trauma in the prone position is a challenge for anaesthetists. Awake fiberoptic nasotracheal intubation, along with airway blocks and appropriate sedation, can be a viable option in such cases. Additionally, successful perioperative treatment relies on effective multidisciplinary cooperation and consultation.

## Data Availability

The datasets used and/or analysed during the current study are available from the corresponding author on reasonable request.
